# Relationship between medication class and ambulatory blood pressure profile in the chronic renal insufficiency cohort (CRIC) study

**DOI:** 10.1038/s41371-026-01166-1

**Published:** 2026-06-06

**Authors:** Sachin V. Pasricha, Debbie L. Cohen, Rachel Shulman, Rushelle Byfield, Jordana B. Cohen, CRIC Study Investigators, Amanda H. Anderson, Lawrence J. Appel, Jing Chen, Debbie L. Cohen, Laura M. Dember, Alan S. Go, L. Lee Hamm, James P. Lash, Mahboob Rahman, Panduranga S. Rao

**Affiliations:** 1 Division of Nephrology, Department of Medicine, University of Toronto, Toronto, ON Canada; 2 Renal-Electrolyte and Hypertension Division, Department of Medicine, Perelman School of Medicine, University of Pennsylvania, Philadelphia, PA USA; 3 Division of Pediatric Nephrology, Department of Pediatrics, Columbia University, New York, NY USA; 4 Department of Biostatistics, Epidemiology, and Informatics, Perelman School of Medicine, University of Pennsylvania, Philadelphia, PA USA; 5 Department of Epidemiology, School of Public Health, The University of Alabama at Birmingham, Birmingham, AL USA; 6 Department of Epidemiology, Bloomberg School of Public Health, Johns Hopkins University, Baltimore, MD USA; 7 Department of Epidemiology, University of Texas Southwestern Medical Center Peter O’Donnell Jr. School of Public Health, Dallas, TX USA; 8 Department of Internal Medicine, University of Texas Southwestern Medical Center, Dallas, TX USA; 9 Renal-Electrolyte and Hypertension Division, Perelman School of Medicine, University of Pennsylvania, Philadelphia, PA USA; 10 Division of Research, Kaiser Permanente Northern California, Pleasanton, CA USA; 11 Department of Medicine, Tulane University School of Medicine, New Orleans, LA USA; 12 Division of Nephrology, Department of Medicine, University of Illinois Chicago, Chicago, IL USA; 13 Department of Medicine, Case Western Reserve University School of Medicine, Cleveland, OH USA; 14 Division of Nephrology, Department of Medicine, University of Michigan, Ann Arbor, MI USA

**Keywords:** Chronic kidney disease, Hypertension

## Abstract

Patients with chronic kidney disease (CKD) are at increased risk for masked and nocturnal hypertension, conditions best identified through ambulatory blood pressure (BP) monitoring (ABPM) and associated with adverse cardiovascular outcomes. We evaluated associations between antihypertensive medication classes and ABPM profiles by conducting a cross-sectional analysis of participants from the Chronic Renal Insufficiency Cohort (CRIC) with ABPM data. Antihypertensive medications were categorized as renin-angiotensin system inhibitors (RASis), beta-blockers, calcium channel blockers (CCBs), and thiazide/loop diuretics. We used multinomial logistic regression to evaluate the independent association between each medication class (accounting for simultaneous use of multiple classes) and ABPM phenotype: controlled hypertension, white coat effect, sustained hypertension, and masked uncontrolled hypertension (MUCH). Secondary outcomes included nocturnal hypertension, nocturnal non-dipping, and BP variability. Analyses were Bonferroni-corrected for multiple comparisons. Among 1499 eligible participants, 66% used RASis, 52% beta-blockers, 43% CCBs, and 50% thiazide/loop diuretics. RASi use was associated with lower odds of sustained hypertension (OR 0.60, 95% CI 0.42 to 0.84), while beta-blocker use was positively associated with MUCH (OR 1.48, 95% CI: 1.12 to 1.96). For secondary outcomes, RASi use was associated with lower odds of nocturnal hypertension (OR 0.71, 95% CI: 0.55 to 0.91), whereas beta-blocker and CCB use were both associated with nocturnal hypertension (OR for beta-blockers 1.33, 95% CI 1.04 to 1.70; OR for CCBs 1.36, 95% CI 1.07 to 1.73). Overall, we identified several associations between specific antihypertensive classes and abnormal ABPM profiles. Longitudinal studies are needed to evaluate reproducibility and potential mechanisms of these findings.

## Introduction

Hypertension is the leading cause of heart attacks, strokes, and cardiovascular mortality, accounting for one in five deaths globally, and the second biggest contributor to chronic kidney disease (CKD) progression [1]. Ambulatory blood pressure (BP) monitoring (ABPM) is the most accurate method to determine discordance between the office and out-of-office BP readings [2]. ABPM is particularly useful in diagnosing masked uncontrolled hypertension (MUCH; i.e., normal office BP with elevated out-of-office BP in individuals with treated hypertension) and white coat effect (i.e., elevated office BP with normal out-of-office BP in individuals with treated hypertension), which may be missed with office-based BP measurements alone in 20 and 30% of adults, respectively [3, 4]. ABPM is also the only mechanism to diagnose other high-risk BP profiles, including nocturnal hypertension, nocturnal non-dipping status, and short-term BP variability [5].

Patients with CKD have a high prevalence of MUCH, nocturnal hypertension, and nocturnal non-dipping status, and these ABPM profiles are strongly associated with an increased risk of cardiovascular events and CKD progression [6]. Most patients with CKD are also treated with antihypertensive medications. Antihypertensive medication classes have variable mechanisms and durations of action, implying that they may impact ABPM phenotype and nocturnal dipping status differently [7]. However, data is lacking on associations between ABPM profiles and different antihypertensive medication classes.

The goal of our study was to evaluate whether individual antihypertensive medication classes are independently associated with specific ABPM profiles in patients with CKD using cross-sectional data from the Chronic Renal Insufficiency Cohort (CRIC) Study. We aimed to generate hypotheses into whether longitudinal assessments of these relationships merit closer examination.

## Methods

### Study population

Our study population comprises participants from the CRIC Study who underwent ABPM and for whom medication data was available at the time of ABPM. The CRIC study enrolled patients aged 21–74 years old enrolled from 2003 to 2008 with an estimated glomerular filtration rate (eGFR) of 20–70 mL/min/1.73 m² who were followed longitudinally thereafter. The CRIC Study excluded individuals with glomerulonephritis, polycystic kidney disease, cirrhosis, class III/IV heart failure, HIV, active infection, active cancer, or pregnancy. Ethical approval was obtained from the institutional review boards at each participating site, and all CRIC Study participants provided written informed consent consistent with the principles outlined in the Declaration of Helsinki.

Between 2008 and 2012, 1502 participants underwent ABPM as part of the study’s second phase. Importantly, the clinical characteristics of participants with ABPM data closely mirror those of the entire CRIC cohort, as demonstrated in previous publications [8].

### Study design

This is a cross-sectional study to assess the independent association between each antihypertensive medication class with ABPM profiles. Importantly, the statistical approach (multinomial logistic regression with penalized *p*-values) accounts for the fact that individuals may have simultaneously been taking multiple medications classes, and the associations studied thereby reflect independent associations between each specific medication class with ABPM profile, accounting for all other antihypertensive medication use [9]. Another possible approach to the issue account for simultaneous use of multiple medication classes would be to evaluate ABPM profiles in the mutually exclusive groups of patients based on combination regimens. However, statistical power would be limited in this approach given the plethora of combination regimens, so we chose the advantageous approach of a multinomial regression with penalized *p*-values.

### Exposure status

Our primary exposure was antihypertensive medication class. Self-reported medication use was collected through questionnaires at each CRIC study visit. We used medication data from the visit corresponding to or immediately preceding ABPM.

We categorized the medication class exposure into four distinct, non-mutually exclusive groups: renin angiotensin system inhibitors (RASis), beta-blockers, calcium channel blockers (CCBs), and thiazide/thiazide-like diuretics or loop diuretics. Individuals may have thereby been exposed to as few as 0, or as many as all 4, medication classes, accounting for potential overlapping medication use. Individuals on fixed dose combination pills (e.g. a combined RASi and CCB) were classified as exposed to both medication classes. In secondary analyses, we expanded medication class exposure to include seven antihypertensive medication classes, categorized as follows: RASis, beta-blockers, CCBs, thiazide/thiazide-like diuretics, loop diuretics, alpha-blockers and potassium-sparing diuretics (which included mineralocorticoid receptor antagonists).

### Covariates

Our primary and secondary analyses were adjusted for baseline eGFR (using the CRIC 2021 equation) [10], urine protein-to-creatinine ratio, sociodemographic data (age, sex, race/ethnicity, household income, education, and current smoking status), comorbidities (diabetes, cardiovascular disease, and heart failure, collected through questionnaires) and body mass index (BMI; measured at each study visit). Missing covariate data was handled using multiple imputation. Notably, there were no missing primary exposure or outcome data as having antihypertensive medication class (primary exposure) data available and ABPM (outcome) data available were required inclusion criteria.

### Primary outcome

The primary outcome was ABPM phenotype categorized into four mutually exclusive groups (i.e., each individual had a single ABPM phenotype) based on a combination of office BP (using the office BP from the visit closest to the ABPM) and ABPM results, as classified by Rahman et al. [6]: controlled hypertension (office BP < 140/90 and mean 24-h ambulatory BP < 130/80), white coat effect (office BP ≥ 140/90 and mean 24-h ambulatory BP < 130/80), sustained hypertension (office BP ≥ 140/90 and mean 24-h ambulatory BP ≥ 130/80), or MUCH (office BP < 140/90 and mean 24-h ambulatory BP ≥ 130/80).

Office BP was measured by trained study staff following the American Heart Association’s standard protocol during all CRIC visits, which involves averaging three seated readings taken after a five-minute rest period. ABPM was performed using SpaceLabs 90207 or 90217 monitors (Snoqualmie, WA, USA). BP readings were taken every 30 min during day and night. To be considered valid, the ABPM required at least 14 daytime readings (6 AM to midnight) and at least six nighttime readings (midnight to 6 AM), as these were commonly accepted validity thresholds when ABPMs were conducted for CRIC [11]. Many societies have different thresholds for ABPM validity thresholds, so we include a sensitivity analysis for our primary and secondary outcomes whereby we only include ABPMs that had at least 20 daytime readings and at least 7 nighttime readings, which is based on the European Society of Hypertension ABPM guidelines [12, 13].

### Secondary outcomes

The secondary outcomes were nocturnal hypertension, nocturnal dipping status, and 24-h BP variability, all derived from the previously described ABPM measurements. Nocturnal hypertension (a binary secondary outcome) was defined as average nighttime systolic BP (SBP) ≥ 120 mmHg and/or average nighttime diastolic BP (DBP) ≥ 70 mmHg. Nocturnal dipping status (another binary secondary outcome) was calculated using the ratio of mean nighttime SBP to mean daytime SBP, classified as dippers (≤0.9) versus non-dippers (>0.9). The 24-h ambulatory SBP and diastolic BP (DBP) variability (separate continuous secondary outcomes) were calculated using average real variability (i.e., the sum of the absolute differences in consecutive ambulatory BP measurements divided by the total number of measurements minus one) [14].

### Statistical plan

Descriptive statistics were used to compare baseline characteristics of individuals exposed to versus unexposed to each medication class, using the chi-square test (for binary or categorical variables), t-test (for normally distributed continuous variables), and Kruskal Wallis test (for non-normally distributed continuous variables). We report the baseline characteristics for individuals exposed to each medication class and the baseline characteristics for the overall population in the main manuscript, and report characteristics of those unexposed to each medication class in supplemental appendices. Any individual exposed to multiple medication classes (e.g. an individual taking a RASi and a beta-blocker) is intentionally included as exposed to both medication classes in the Table summarizing baseline characteristics in the main manuscript (Table 1).

**Table 1:** Baseline characteristics by medication class.

	Overall	RASis	Beta-blockers	CCBs	Thiazide or loop diuretics
**N (%)**	1 499	984 (66%)	777 (52%)	638 (43%)	756 (50%)
**Age, years (mean **± **SD)**	63.1 ± 10.3	63.3 ± 10.1	64.8 ± 9.2*	64.4 ± 9.6*	64.8 ± 9.2*
**Female Sex, N (%)**	661 (44.1%)	390 (40%)*	326 (42%)	266 (42%)	333 (44%)
**Race/Ethnicity, N (%)**			*		*
*White*	679 (45.3%)	433 (44%)	317 (41%)	238 (37%)	284 (38%)
*Black*	581 (38.8%)	387 (39%)	339 (44%)	302 (47%)	343 (45%)
*Hispanic*	182 (12.1%)	118 (12%)	96 (12%)	72 (11%)	103 (14%)
*Other*	57 (3.8%)	46 (5%)	25 (3%)	26 (4%)	26 (3%)
**BMI, kg/m**^**2**^ **(mean **± **SD)**	31.5 ± 7.0	32.0 ± 6.8*	32.4 ± 6.9*	31.9 ± 6.9	32.3 ± 6.9*
**Education, N (%)**			*	*	*
*Less than HS*	250 (16.7%)	150 (15%)	141 (18%)	122 (19%)	150 (20%)
*HS graduate*	259 (17.3%)	174 (18%)	167 (21%)	117 (18%)	138 (18%)
*Some college*	468 (31.2%)	315 (32%)	248 (32%)	214 (34%)	249 (33%)
*College grad or greater*	521 (34.8%)	345 (35%)	221 (28%)	185 (29%)	219 (29%)
*Unknown*	1 (0.1%)	0 (0%)	0 (0%)	0 (0%)	0 (0%)
**Income, N (%)**		*	*	*	*
*≤ $20,000*	393 (26.2%)	240 (24%)	243 (31%)	178 (28%)	233 (31%)
*$20,001–50,000*	401 (26.8%)	263 (27%)	211 (27%)	189 (30%)	207 (27%)
*$50,001–100,000*	334 (22.3%)	230 (23%)	145 (19%)	123 (19%)	139 (18%)
*≥ $100,001*	163 (10.9%)	119 (12%)	72 (9%)	54 (8%)	67 (9%)
*Did not answer*	208 (13.9%)	132 (13%)	106 (14%)	94 (15%)	110 (15%)
**Insurance, N (%)**			*	*	*
*None*	115 (7.7%)	68 (7%)	70 (9%)	57 (9%)	65 (9%)
*Any Public*	786 (52.4%)	512 (52%)	456 (59%)	366 (57%)	447 (59%)
*Private*	250 (16.7%)	161 (16%)	101 (13%)	77 (12%)	100 (13%)
*Unknown*	348 (23.2%)	243 (25%)	150 (19%)	138 (22%)	144 (19%)
**Illicit drug use, N (%)**	505 (33.7%)	318 (32%)	248 (32%)	221 (35%)	241 (32%)
**Current smoker, N (%)**	131 (8.7%)	69 (7%)*	70 (9%)	69 (11%)*	67 (9%)
**eGFR, mL/min/1.73 m**^**2**^ **(mean **± **SD)**	46.1 ± 20.2	45.3 ± 17.8*	41.7 ± 18.2*	42.0 ± 19.3*	40.3 ± 17.7*
**Urine protein-to-creatinine ratio, mg/mg (median [IQR])**	0.15 (0.07–0.60)	0.15 (0.07–0.55)	0.19 (0.08–0.80)*	0.26 (0.09–0.96)*	0.18 (0.08–0.76)*
**Hypertension, N (%)**	1394 (93.0%)	975 (99%)*	768 (99%)*	637 (99.8%)*	750 (99%)*
**Number of antihypertensive medications, (median [IQR])**	3 (1–4)	3 (2–4)*	3 (3–4)*	3 (3–4)*	3 (3–4)*
**Diabetes, N (%)**	742 (49.5%)	542 (55%)*	450 (58%)*	348 (55%)*	472 (62%)*
**Cardiovascular disease, N (%)**	584 (39.0%)	409 (42%)*	423 (54%)*	289 (45%)*	362 (48%)
**Congestive heart failure, N (%)**	146 (9.7%)	96 (10%)	119 (15%)*	67 (10%)	115 (15%)
**Atrial fibrillation, N (%)**	292 (19.5%)	197 (20%)	201 (26%)*	149 (23%)*	190 (25%)
**Dyslipidemia, N (%)**	1385 (92.4%)	946 (96%)*	739 (95%)*	604 (95%)*	725 (96%)

Most continuous variables were compared using a t-test of means with a two-sided alpha significance of 0.05, with the exception of urine protein-to-creatinine ratio and number of antihypertensive medications, which were compared with a Kruskal-Wallis equality of proportions rank test with a two-sided alpha significance of 0.05. The proportion of people on each medication class versus not with each categorical and binary baseline characteristic were compared using a chi-squared test with a significance level of 0.05.

*BMI* body mass index, *CCBs* calcium channel blockers, *eGFR* estimated glomerular filtration rate, *HS* high school, *IQR* interquartile range, *RASis* renin angiotensin system inhibitors, *SD* standard deviation.

*Indicates that there is a significant difference for this baseline characteristic comparing the group of individuals on the medication class (displayed in the table) vs. those not on the medication class (not displayed on the table; for details, see the supplementary appendix Tables 1–4.

Separate multinominal logistic regression models were used to evaluate for an independent association between each medication class and specific ABPM phenotypes for our primary analysis, adjusted for the covariates described above (using log-transformations for any non-normally distributed covariates). Similarly, separate adjusted logistic regression models were used to evaluate for an independent association between each medication class and dichotomous secondary outcomes (nocturnal hypertension and nocturnal dipping status), while separate adjusted linear regression models were used to evaluate for an independent association between each medication class and continuous secondary variables (24-h SBP and DBP variability). A Bonferroni-corrected alpha of 0.0125 was considered statistically significant for the primary analysis to account for multiple comparisons.

## Results

### Baseline characteristics

**Fig. 1:**
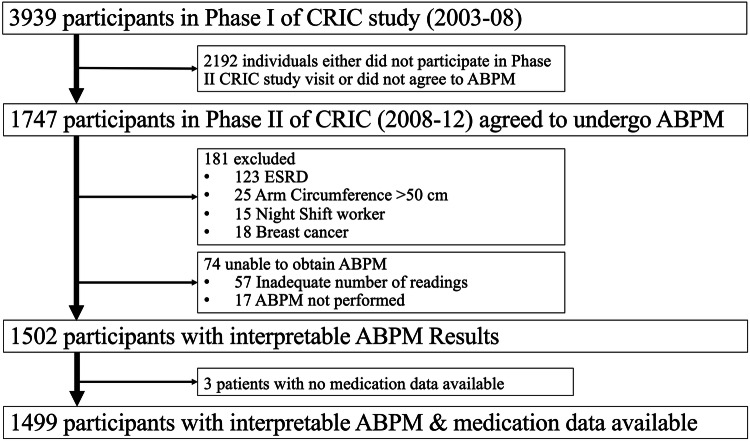
Description of cohort identification. Phase I of CRIC enrolled 3939 patients, of whom 1747 agreed to ABPM as a part of Phase II. Of these, 1502 individuals had interpretable ABPMs. Prior CRIC studies describe 1502 participants who had ABPM readings in CRIC Phase II (2008–2012) [6]. This figure further details how these 1502 participants were narrowed to the 1499 participants included in the present study (i.e. after excluding 3 patients with missing medication data).

Our study population included 1499 of the 1502 individuals with CKD in the CRIC study who had ABPM (Fig. 1). The median number of medications was 3 (IQR: 1–4), with RASis being most common (66%), followed by beta-blockers (52%), thiazide/loop diuretics (50%), and CCBs (43%) (Table 1, Tables S1-S4, & Table S5-9**for sensitivity analysis**). Mean age was 63.1 ± 10.3 years and 44.1% of participants were female. Individuals on beta-blockers, CCBs, and thiazide/loop diuretics were less likely to be of college education or higher, and more likely to be of the lowest income quintile. Individuals who were prescribed RASis were least likely to currently smoke (7%).

### Prevalence of ABPM outcomes

Of the 1499 included individuals, 740 (49%) had an ABPM phenotype of controlled hypertension, while 430 (29%) had MUCH, 268 (18%) had sustained hypertension, and 61 (4%) had white coat effect. With respect to nocturnal BP secondary outcomes, 821 (55%) individuals had nocturnal hypertension and 823 (55%) individuals had nocturnal non-dipping. The average SBP variability was 10.21 (±2.47) mmHg and the average DBP variability was 7.38 (±1.72) mmHg for the entire cohort.

### Prevalence of each antihypertensive medication class combination

Our statistical approach accounts for overlapping use of multiple medication classes. Table 2 reports the prevalences of different combinations of antihypertensive medication classes. RASi monotherapy (*N* = 178; 12%) was the most common combination, followed by triple therapy with a RASi, beta-blocker, and thiazide/loop diuretic (*N* = 167; 11%) and quadruple therapy with a RASi, beta-blocker, CCB, and thiazide/loop diuretic (*N* = 154; 10%).

**Table 2:** Prevalence of each combination of anti-hypertensive medication classes.

Combination of antihypertensive medication classes	N (%)
No antihypertensive medications	139 (9%)
RASi	178 (12%)
Beta-blocker	70 (5%)
CCB	35 (2%)
Thiazide/loop diuretic	33 (2%)
RASi + beta-blocker	101 (7%)
RASi + CCB	94 (6%)
RASi + thiazide/loop diuretic	94 (6%)
Beta-blocker + CCB	38 (3%)
Beta-blocker + thiazide/loop diuretic	79 (5%)
CCB + thiazide/loop diuretic	41 (3%)
RASi + beta-blocker + CCB	88 (6%)
RASi + beta-blocker + thiazide/loop diuretic	167 (11%)
RASi + CCB + thiazide/loop diuretic	108 (7%)
Beta-blocker + CCB + thiazide/loop diuretic	80 (5%)
RASi + beta-blocker + CCB + thiazide/loop diuretic	154 (10%)

*CCB* calcium channel blocker, *RASi* renin angiotensin system inhibitor.

### Unadjusted relationship of antihypertensive classes with office BP and ABPM

Individuals on beta-blocker-, CCB-, or thiazide/loop diuretic-based regimens had elevated office SBPs, compared to those on alternative regimens, whereas individuals on RASi- beta-blocker-, and thiazide/loop diuretic-based regimens had lower office DBPs, compared to those on alternative regimens (Table 3, Table S10**for** ***p*-values, &** Table S11**for sensitivity analysis**). Similarly, the 24-h average SBP on ABPM was elevated in individuals on beta-blockers, CCBs, and thiazide/loop diuretics, compared to those not on these medication classes, and 24-h average SBP was lower in those on RASis, compared to those not on RASis. The 24-h average DBP on ABPM was also lower in individuals on RASis, beta-blockers, and thiazide/loop diuretics, compared to those not on these medication classes.

**Table 3:** Baseline office and 24-h ambulatory blood pressures by medication class.

Mean, mmHg ± SD	Overall	RASis	Beta-blockers	CCBs	Thiazide or loop diuretics
Office SBP	126.2 ± 20.3	125.5 ± 19.6	128.4 ± 21.2*	129.3 ± 19.2*	128.4 ± 20.8*
Office DBP	69.2 ± 12.2	68.5 ± 12.1*	68.5 ± 12.7*	68.7 ± 12.3	67.7 ± 12.7*
24-hr SBP Average	128.4 ± 16.0	127.7 ± 15.4*	130.4 ± 16.3*	131.7 ± 14.9*	130.1 ± 15.9*
24 h DBP Average	72.4 ± 9.6	71.6 ± 9.4*	71.9 ± 10.0*	72.8 ± 9.5	71.2 ± 9.9*
Daytime SBP Average	132.2 ± 15.7	131.5 ± 15.2*	133.6 ± 16.2*	134.9 ± 14.5*	133.5 ± 15.7*
Daytime DBP Average	75.7 ± 10.0	74.8 ± 9.8*	74.7 ± 10.3*	75.6 ± 9.7	74.1 ± 10.2*
Nighttime SBP Average	120.9 ± 19.1	120.2 ± 18.3*	124.1 ± 19.6*	125.3 ± 18.5*	123.5 ± 19.1*
Nighttime DBP Average	66.6 ± 10.9	65.9 ± 10.6*	66.7 ± 11.6	67.7 ± 11.0*	65.9 ± 11.3*

*CCBs* calcium channel blockers, *DBP* diastolic blood pressure, *RASis* renin angiotensin system inhibitors, SBP systolic blood pressure, *SD* standard deviation.

*Indicates that there is a significant difference for this blood pressure characteristic comparing the group of individuals on the medication class (displayed in the table) vs. those not on the medication class (not displayed on the table; for details, see the supplementary appendix Table S10) using a two-sample t-test with a two-sided alpha of 0.05.

### Adjusted association of antihypertensive classes with ABPM phenotypes (primary outcome)

**Fig. 2:**
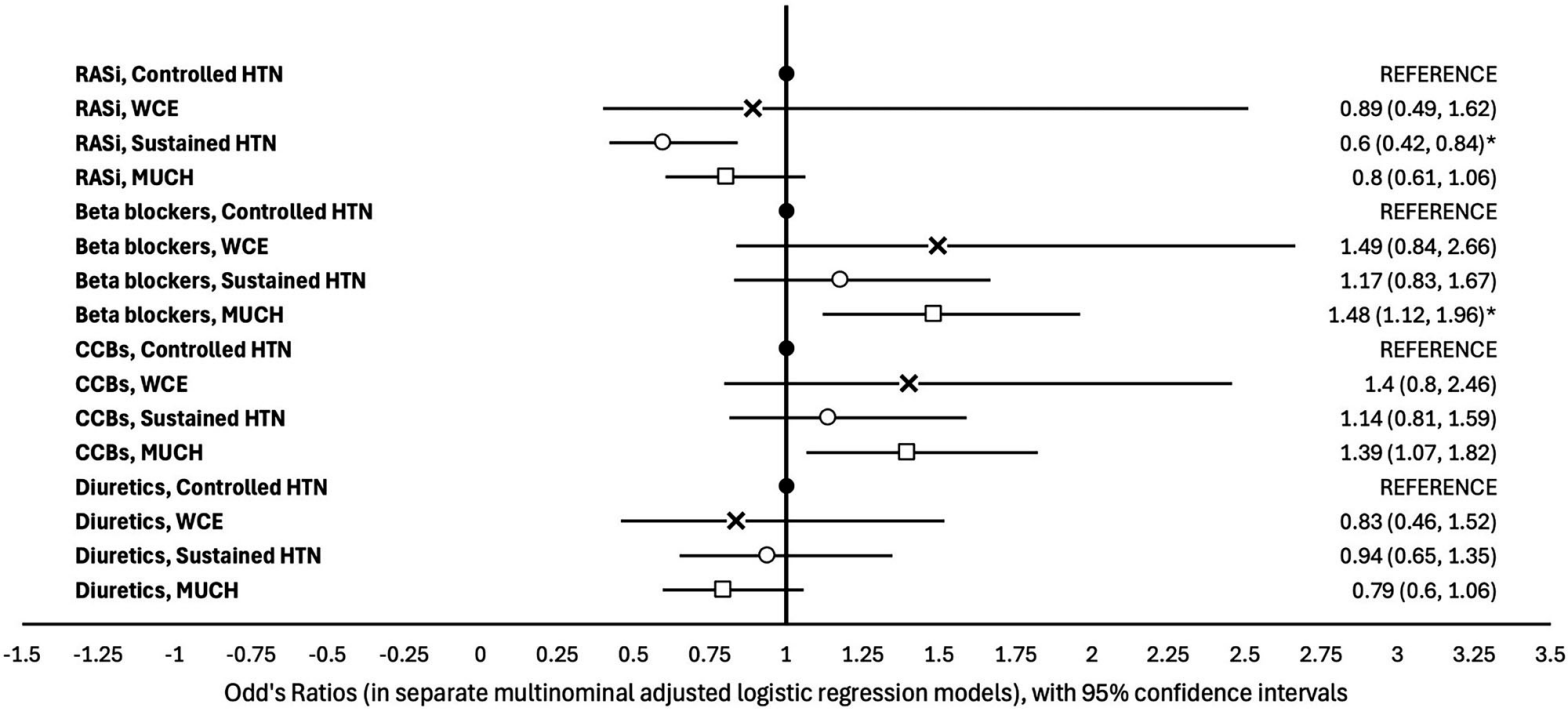
Forest plot of primary outcome analysis. Figure 2 displays the results of separate adjusted multinominal logistic regression analyses to investigate the independent association of each medication class with each ABPM phenotype. Adjustments were made for the following covariates: baseline estimated glomerular filtration rate (using the CRIC 2021 equation) (10), urine protein-to-creatinine ratio (with log transformation), sociodemographic data (age, sex, race/ethnicity, household income, education, and smoking status), comorbidities (diabetes, cardiovascular disease, and heart failure) and body mass index. * indicates significance using a Bonferroni corrected alpha of 0.0125. ● indicates odds ratio for having controlled hypertension for each specific medication class (i.e. reference). X indicates odds ratio for having sustained hypertension for each specific medication class.○ indicates odds ratio for having white coat effect for each specific medication class. □ indicates odds ratio for having masked uncontrolled hypertension for each specific medication class.

Our primary analysis was to evaluate the association between antihypertensive medication class and ABPM phenotype (Fig. 2, Table S12**for** ***p*-values, &** Table S13**for sensitivity analysis**). RASi use was independently associated with lower odds of sustained hypertension (odds ratio [OR] 0.60, 95% CI 0.42 to 0.84, *p* = 0.003), while beta-blocker use was independently associated with higher odds of MUCH (OR 1.48, 95% CI: 1.12 to 1.96, *p* = 0.006). No specific medication class was associated with white coat effect or controlled hypertension. A sensitivity analysis that included additional medication classes also demonstrated that loop diuretic use was independently associated with lower odds of white coat effect (OR 0.48, 95% CI: 0.24 to 0.95, *p* = 0.035) (Table S11).

### Adjusted association of antihypertensive classes with other high-risk ABPM profiles (secondary outcomes)

For our secondary outcomes (Table 4, Table S14**for** ***p*-values, &** Table S15**for sensitivity analysis**), RASi use was independently associated with lower odds of nocturnal hypertension (OR: 0.71, 95% CI: 0.55 to 0.91, *p* = 0.008), but beta-blocker use and CCB use were both independently associated with higher odds of nocturnal hypertension (OR for beta-blockers: 1.33, 95% CI 1.04 to 1.70, *p* = 0.024; OR for CCBs: 1.36, 95% CI 1.07 to 1.73, *p* = 0.011). Beta-blocker use and CCB use were also both independently associated with nocturnal non-dipping (OR for beta-blockers: 1.29, 95% CI 1.03 to 1.64, *p* = 0.028; OR for CCBs: 1.26, 95% CI 1.01 to 1.59, *p* = 0.042). Thiazide/loop diuretic use was not independently associated with any high-risk ABPM profile. Finally, CCB use was also independently associated with lower 24-h SBP variability (β: −0.34, CI: −0.59 to −0.10, *p* = 0.007) and lower 24-h DBP variability (β: −0.34, CI: −0.51 to −0.16, *p* < 0.0001).

**Table 4:** Secondary (nocturnal blood pressure and blood pressure variability) outcomes, by medication class.

	RASis	Beta-blockers	CCBs	Thiazide or loop diuretics
Nocturnal blood pressure (secondary outcomes)				
Nocturnal HTN (SBP ≥ 120 mmHg or DBP ≥ 70 mmHg), OR (95% CI)	0.71	1.33	1.36	0.94
	(0.55, 0.91) *	(1.04, 1.70) *	(1.07, 1.73) *	(0.73, 1.21)
Non-dipping	1.01	1.29	1.26	1.09
OR (95% CI)	(0.80, 1.28)	(1.03, 1.64) *	(1.01, 1.59) *	(0.86, 1.39)
Blood pressure variability (secondary outcomes)				
24-h systolic variability,	0.03	−0.11	−0.34	0.06
β coefficient (95% CI)	(−0.22, 0.29)	(−0.36, 0.15)	(−0.59, −0.10) *	(−0.20, 0.33)
24-h diastolic variability,	0	−0.11	−0.34	0.04
β coefficient (95% CI)	(−0.18, 0.18)	(−0.29, 0.07)	(−0.51, −0.16) *	(−0.14, 0.23)

Adjustments were made for the following covariates: baseline estimated glomerular filtration rate (using the CRIC 2021 equation) (10), urine protein-to-creatinine ratio (with log transformation), sociodemographic data (age, sex, race/ethnicity, household income, education, and smoking status), comorbidities (diabetes, cardiovascular disease, and heart failure) and body mass index.

*β* beta, *CCBs* calcium channel blockers, *CI* confidence interval, *HTN* hypertension, *OR* odds ratio, *RASis* renin angiotensin system inhibitors.

*Denotes statistically significant values using an alpha of 0.05 (for secondary outcome analyses). *P*-values for all comparisons are presented in the Supplemental Appendix Tables S12 and S14.

A sensitivity analysis (Table S15) that included additional medication classes demonstrated that thiazide diuretic use was independently associated with lower 24-h SBP variability (β: 0.37, 95% CI 0.06 to 0.67, *p* = 0.017) and lower 24-h DBP variability (β: 0.23, 95% CI 0.02 to 0.45, *p* = 0.036). Alpha blocker use was independently associated with higher odds of nocturnal hypertension (OR 1.61, 95% CI 1.17 to 2.22, *p* = 0.004), higher odds of nocturnal non-dipping (OR 1.48, 95% CI 1.10 to 2.01, *p* = 0.010), and lower 24-h DBP variability (β: −0.27, 95% CI −0.49 to −0.04, *p* = 0.020).

A separate sensitivity analysis for the primary and secondary outcomes using only 1440 individuals who had ABPMs with at least 20 daytime readings and at least 7 nighttime readings demonstrated similar results (Table S16).

## Discussion

Our study investigated associations between four major classes of antihypertensive medications and ABPM profiles in patients with CKD. While many individuals were on combinations of antihypertensive medication classes, our statistical approach enabled assessing the independent association of each medication class with ABPM profiles. In the primary analysis, RASi use was associated with lower odds of sustained hypertension, whereas beta-blocker use was associated with higher odds of MUCH. In secondary analyses, we identified that RASi use was associated with lower odds of nocturnal hypertension while both beta-blocker and CCB use were independently associated with higher odds of nocturnal hypertension and non-dipping status. Additionally, CCB use was associated with lower BP variability. These findings represent potential links between antihypertensive medication classes and several high-risk ABPM profiles that cannot be identified using office BP alone.

Our primary objective was to assess the relationship between antihypertensive medication class and ABPM phenotype. While prior research has thoroughly explored demographic and clinical predictors of MUCH, including diabetes, male sex, smoking, and borderline elevated office BPs, few studies have addressed pharmacologic exposures in this context [3, 15]. For instance, the International Database on Ambulatory BP in Relation to Cardiovascular Outcomes (IDACO), which included nearly 10,000 participants from 11 countries, identified diabetes as a significant risk factor for masked hypertension (29% prevalence of masked hypertension among individuals with diabetes vs. 19% among individuals without diabetes) [3]. Similarly, the Jackson Heart Study, comprising nearly 1000 African American participants, found that male sex (OR 1.74, 95% CI 1.18–2.56), smoking (OR 1.93, 95% CI 1.06–3.52), and office BP (OR 4.21, 95% CI 1.33–13.36 for BP 130–139/85–89 mm Hg) were associated with higher odds of masked hypertension [15]. Obesity, interestingly, was not significantly associated with masked hypertension in this study, but has been in prior studies, such as a Chinese cohort of nearly 3000 participants [16]. The Jackson Heart Study also observed that use of antihypertensive medications was associated with MUCH (OR 1.61, 95% CI 1.07–2.43), though did not evaluate specific medication classes. Our study contributes to this area of study by identifying significant, independent associations between use of specific antihypertensive classes (namely beta-blockers) and the MUCH phenotype.

Nocturnal hypertension was a secondary outcome of interest in our study. Existing literature underscores the high prevalence and clinical importance of nocturnal hypertension, particularly in individuals with diabetes, among whom rates of nocturnal non-dipping and nocturnal hypertension can approach 65% [17]. In patients with diabetes, risk factors for uncontrolled nocturnal hypertension include obesity (OR 1.6, 95% CI 0.9–1.8), duration of diabetes > 10 years (OR 1.4, 95% CI 0.9–2.4), and age > 65 years old (OR 1.9, 95% CI 1.2–3.1) [18]. Importantly, isolated nocturnal hypertension is associated with higher mortality risk (HR 1.29, 95% CI 1.01–1.65), stressing the critical role of nocturnal BP assessment in diagnostic and therapeutic decisions [19]. In prior studies of patients with both CKD and hypertension, one-third have uncontrolled nocturnal hypertension, but to our knowledge, no prior investigation has evaluated the association between specific medication classes and nocturnal hypertension [20].

Our study is cross-sectional and thus cannot infer causality, directionality, or mechanisms of the relationships between antihypertensive classes and ABPM profiles, particularly in the absence of granular data on medication timing, frequency, half-life, dosing, or within-class differences. However, our goal was to distinctively generate hypotheses with respect to the relationship between entire antihypertensive medication classes and ABPM profiles in patients with CKD. First, the inverse relationship of RASi use with sustained may stem from how RAS pathway overactivation is a primary driver of hypertension in CKD, and inhibiting this pathway reduces the odds of sustained hypertension [21]. Similarly, the RAS pathway has been implicated as a mechanistic contributor to isolated nocturnal hypertension, which could explain why RASi use was associated with reduced odds of nocturnal hypertension [22]. Alternatively, this inverse association may reflect preferential use of RASis as first-line anti-hypertensive monotherapy in patients with CKD and mild hypertension due to their cardiovascular and antiproteinuric benefits (as is reflected by RASi monotherapy being the most common medication regimen used in our study population) [23–25]. The presumed milder nature of hypertension in many patients on RASis would consequentially account for our finding that RASi use was associated with lower odds of both sustained and nocturnal hypertension.

Second, we identified higher odds of MUCH in patients on beta-blockers. A proposed mechanism is that these medications inhibit the sympathetic nervous system and exhibit anxiolytic effects thus blunting the common rise in BP that occurs in office settings, leading to office BPs below office thresholds for hypertension, but ambulatory BPs that exceed ambulatory thresholds [26]. Alternatively, individuals with MUCH may have been preferentially prescribed beta-blockers because clinicians perceived them as having higher cardiovascular risk for other reasons that are also known to be associated with MUCH and were not captured by our data, such as prior smoking history (as opposed to current smoking status, which we were able to adjust for in our analyses). Third, we identified associations between CCB use and both nocturnal hypertension and non-dipping. Nocturnal hypertension has been hypothesized to be primarily volume- and sympathetically-mediated [27], which may explain why CCB-based vasodilation has a less impactful effect on nocturnal BPs. Prior observational studies that found amlodipine and clindapine (both CCBs) increase nocturnal BP in extreme nocturnal dippers, further supporting our findings [28, 29]. Conversely, providers may have preferentially prescribed CCBs to patients with overlapping risk factors for nocturnal non-dipping. Reduced sympathetic tone and enhanced vagal tone are key contributors to nocturnal BP dipping, which mechanistically suggests that beta blockers, which achieve both effects, should reduce nocturnal hypertension and non-dipping [22]. Paradoxically, we found that beta blockers were inversely associated with nocturnal hypertension. Our finding may reflect indication-based prescribing of beta blockers to individuals with congestive heart failure and cardiovascular disease, conditions which were more common in individuals exposed to beta blockers and have been associated with nocturnal hypertension [27]. Ultimately, our identified associations of RASi use with lower odds of sustained and nocturnal hypertension, beta-blocker use with MUCH, and CCB use with nocturnal hypertension/non-dipping generate several hypotheses that warrant further evaluation.

Our study’s primary strength is its novelty, being the first, to our knowledge, to evaluate whether specific antihypertensive medication classes are associated with ABPM phenotypes. The second major strength is the detailed, systematic nature of the data prospectively collected in CRIC, allowing us to adjust for several important sociodemographic and clinical covariates. Third, our primary statistical approach, a multinomial regression with penalized *p*-values, enabled us to account for the fact that antihypertensive therapy is commonly not mutually exclusive, wherein multiple drug classes are frequently prescribed in combination. In this setting, multinomial regression is advantageous because it permits comparison across multiple outcome categories within a single coherent model while appropriately adjusting for correlated, non-mutually exclusive exposures. Antihypertensive drug classes were modeled as separate indicator variables that could be applied simultaneously, with the use of any given drug class conditional on concurrent use of other classes. This avoided the forced attribution of patients to a single treatment category that would obscure real-world combination therapy.

Our study has several notable limitations. First, as noted previously, the cross-sectional design means we cannot infer causality, temporality, or mechanisms for our findings, and some findings may be the result of confounding by indication. Second, due to the large number of comparisons, we did not perform stratified analyses by potential effect modifiers, potentially obscuring subgroup-specific associations (e.g., diuretics contributing to nocturnal hypertension in obese patients). High-risk subgroups will be evaluated in future manuscripts. Third, our analysis is limited to patients with CKD in the CRIC study and thus cannot be generalized to the broader population or individuals with causes of CKD excluded from CRIC (i.e. polycystic kidney disease, glomerulonephritis, heart failure). Fourth, we lack data on medication adherence, dosage, frequency, timing, and half-life (i.e. cannot differentiate between short- and long-acting medications), all of which may relate to the relationship of a specific medication class with ABPM profiles. Fifth, we intentionally did not adjust for number of antihypertensive medications. RASi monotherapy was the most common first- and second-line medication (shown in Table 2) so adjusting for number of antihypertensive medications was collinear and could inappropriately reduce the effect size of RASi on ABPM profiles. Sixth, we combined thiazide and loop diuretics in our primary analysis, as thiazide diuretics are often switched to loop diuretics as CKD progresses, which was the case in our cohort (Table S5) [30]. We did evaluate these as distinct classes in our secondary analyses. Seventh, we adjust based on urine protein to creatinine ratio instead of urine albumin to creatinine ratio given we had more complete data for this baseline characteristic, though urine albumin to creatinine ratio is now more commonly used in clinical practice. Eighth, we collapsed the four traditional nocturnal dipping status outcomes, extreme dippers (≤0.9), dippers (>0.8 and ≤ 0.9), non-dippers (>0.9 and ≤ 1.0) and reverse dippers (>1.0), into two categories, dippers (≤0.9) and non-dippers (>0.9), to streamline our analysis, but this may have affected our findings. We also used fixed time intervals to reflect nighttime (midnight to 6 AM) and daytime (6 AM to midnight), which may lead to misclassification bias if individuals of different ages, occupations, and cultures have different sleep habits. We did exclude nightshift workers to avoid excessive misclassification of daytime and nighttime status. Finally, recent guidelines suggest lower BP thresholds, office/home BP ≥ 130/80 (as opposed to ≥ 140/90), which corresponds to 24-h ambulatory BP ≥ 125/75 (as opposed to ≥ 130/80), which could change outcome classification [31]. We intentionally chose to use BP thresholds from when these ABPMs were conducted (2008 to 2012) as clinicians at that time were titrating treatment to these higher BP thresholds.

In summary, we identified that several antihypertensive medication classes are independently associated with abnormal ABPM profiles. There are numerous mechanisms that could explain these relationships. Future longitudinal investigations are warranted to determine the reproducibility and directionality of these relationships.

## Summary table

### What is known about the topic

- Ambulatory blood pressure (BP) monitoring (ABPM) profiles of masked uncontrolled hypertension (MUCH) (i.e. elevated out-of-office BP with normal in-office BP) and nocturnal hypertension are associated with increased cardiovascular risk and chronic kidney disease (CKD) progression among patients with CKD.
- Certain comorbidities (such as diabetes and obesity) have been linked to MUCH, but to our knowledge, there is no data on the association of specific antihypertensive medication classes with ABPM profiles.
- Antihypertensive medications have variable mechanisms that may impact ABPM profiles differently, yet this has not yet been evaluated.

### What this study adds

- In cross-sectional analyses, we observed independent associations between several antihypertensive medication classes (accounting for simultaneous use of multiple classes) and ABPM profiles in a cohort of 1499 patients with CKD.
- Renin-angiotensin system inhibitor use was associated with lower odds of sustained hypertension and nocturnal hypertension, while beta-blocker use was associated with higher odds of MUCH and nocturnal hypertension.

## Supplementary information

Supplemental Materials

## Data Availability

Data are publicly available upon request with appropriate regulatory approvals from the National Institute of Diabetes and Digestive and Kidney Diseases Central Repository at https://repository.niddk.nih.gov/home.
