# Supplementary material for: Relationship between medication class and ambulatory blood pressure profile in the chronic renal insufficiency cohort (CRIC) study
Source: J Hum Hypertens. 2026 Jun 6;40(8):631–9. doi: 10.1038/s41371-026-01166-1 (PMC13322747; doi:10.1038/s41371-026-01166-1)
Supplement: Supplementary file 1 [file 41371_2026_1166_MOESM1_ESM.docx]

**Supplementary Appendix for “Relationship Between Medication Class and Ambulatory Blood Pressure Profile in the Chronic Renal Insufficiency Cohort (CRIC)”**

[Table S1. Baseline characteristics of individuals on Renin Angiotensin System inhibitors (RASis) vs. those not on RASis.	2](#_Toc224062224)

[Table S2. Baseline characteristics of individuals on beta blockers vs. those not on beta blockers.	3](#_Toc224062225)

[Table S3. Baseline characteristics of individuals on calcium channel blockers (CCBs) vs. those not on CCBs.	4](#_Toc224062226)

[Table S4. Baseline characteristics of individuals on thiazide/loop diuretics vs. those not on thiazide/loop diuretics.	5](#_Toc224062227)

[Table S5. Sensitivity analysis: Baseline characteristics for additional medication classes.	6](#_Toc224062228)

[Table S6. Sensitivity analysis: Baseline characteristics of individuals on thiazide diuretics vs. those not on thiazide diuretics.	8](#_Toc224062229)

[Table S7. Sensitivity analysis: Baseline characteristics of individuals on loop diuretics vs. those not on loop diuretics.	9](#_Toc224062230)

[Table S8. Sensitivity analysis: Baseline characteristics of individuals on alpha blockers vs. those not on alpha blockers.	10](#_Toc224062231)

[Table S9. Sensitivity analysis: Baseline characteristics of individuals on potassium-sparing diuretics vs. those not on potassium-sparing diuretics.	11](#_Toc224062232)

[Table S10. Baseline office and 24-hour ambulatory blood pressure among those using and not using each medication class.	12](#_Toc224062233)

[Table S11. Sensitivity analysis: Baseline office and 24-hour ambulatory blood pressure of those using and not using each additional medication classes.	13](#_Toc224062234)

[Table S12. P-values for ambulatory blood pressure phenotype by medication class: (Table 4)	14](#_Toc224062235)

[Table S13. Sensitivity analysis: Ambulatory blood pressure phenotype by medication class, including additional medication classes.	15](#_Toc224062236)

[Table S14. P-values for nocturnal blood pressure and blood pressure variability analyses (secondary outcomes, Table 4)	16](#_Toc224062237)

[Table S15. Sensitivity analysis: Nocturnal blood pressure and blood pressure variability (secondary outcomes) by medication class, including additional medication classes.	17](#_Toc224062238)

[Table S16. Sensitivity analysis: Primary and secondary outcome analysis, using alternative threshold for ambulatory blood pressure validity.	18](#_Toc224062239)

## **Table S1. Baseline characteristics of individuals on Renin Angiotensin System inhibitors (RASis) vs. those not on RASis.**

|  | **RASis** | **No RASis** | **p-value** |
| --- | --- | --- | --- |
| **N (%)** | 984 (66) | 515 (34) |  |
| **Age, years (mean** ± **SD)** | 63.3 ± 10.1 | 62.6 ± 10.6 | 0.245 |
| **Female Sex, N (%)** | 390 (40) | 271 (53) | <0.001 |
|  |  |  |  |
| **Race/Ethnicity, N (%)** |  |  |  |
| *White* | 433 (44) | 246 (48) | 0.069 |
| *Black* | 387 (39) | 194 (38) |  |
| *Hispanic* | 118 (12) | 64 (12) |  |
| *Other* | 46 (5) | 11 (2) |  |
| **BMI, kg/m^2^ (mean** ± **SD)** | 32.0 ± 6.8 | 30.5 ± 7.2 | <0.001 |
| **Education, N (%)** |  |  |  |
| *Less than HS* | 150 (15) | 100 (19) | 0.174 |
| *HS graduate* | 174 (18) | 85 (16) |  |
| *Some college* | 315 (32) | 153 (30) |  |
| *College grad or greater* | 345 (35) | 176 (34) |  |
| *Unknown* | 0 (0) | 1 (0.2) |  |
| **Income, N (%)** |  |  |  |
| *≤ $20,000* | 240 (24) | 153 (30) | 0.049 |
| *$20,001-50,000* | 263 (27) | 138 (27) |  |
| *$50,001-100,000* | 230 (23) | 104 (20) |  |
| *≥ $100,001* | 119 (12) | 44 (9) |  |
| *Did not answer* | 132 (13) | 76 (15) |  |
| **Insurance, N (%)** |  |  |  |
| *None* | 68 (7) | 47 (9) | 0.163 |
| *Any Public* | 512 (52) | 274 (53) |  |
| *Private* | 161 (16) | 89 (17) |  |
| *Unknown* | 243 (25) | 105 (20) |  |
| **Illicit drug use, N (%)** | 318 (32) | 187 (36) | 0.120 |
| **Current smoker, N (%)** | 69 (7) | 62 (12) | 0.001 |
| **eGFR, mL/min/1.73m^2^ (mean** ± **SD)** | 45.3 ± 17.8 | 47.6 ± 24.1 | 0.040 |
| **Urine protein-to-creatinine ratio, mg/mg (median [IQR])** | 0.15 (0.07-0.55) | 0.17 (0.07-0.70) | 0.343 |
| **Hypertension, N (%)** | 975 (99) | 419 (81) | <0.001 |
| **Number of antihypertensive medications, (median [IQR])** | 3 (2-4) | 2 (1-3) | <0.001 |
| **Diabetes, N (%)** | 542 (55) | 200 (39) | <0.001 |
| **Cardiovascular disease, N (%)** | 409 (42) | 175 (34) | 0.004 |
| **Congestive heart failure, N (%)** | 96 (10) | 50 (10) | 0.997 |
| **Atrial fibrillation, N (%)** | 197 (20) | 95 (18) | 0.465 |
| **Dyslipidemia, N (%)** | 946 (96) | 439 (85) | <0.001 |

Abbreviations: BMI, body mass index; eGFR, estimated glomerular filtration rate; HS, high school; IQR, interquartile range; RASis, renin angiotensin system inhibitors; SD, standard deviation.

## **Table S2. Baseline characteristics of individuals on beta blockers vs. those not on beta blockers.**

|  | **Beta blockers** | **No beta blockers** | **p-value** |
| --- | --- | --- | --- |
| **N (%)** | 777 (52) | 722 (48) |  |
| **Age, years (mean** ± **SD)** | 64.8 ± 9.2 | 61.2 ± 11.0 | <0.001 |
| **Female Sex, N (%)** | 326 (42) | 335 (46) | 0.083 |
| **Race/Ethnicity, N (%)** |  |  |  |
| *White* | 317 (41) | 362 (50) | <0.001 |
| *Black* | 339 (44) | 242 (34) |  |
| *Hispanic* | 96 (12) | 86 (12) |  |
| *Other* | 25 (3) | 32 (4) |  |
| **BMI, kg/m^2^ (mean** ± **SD)** | 32.4 ± 6.9 | 30.5 ± 6.9 | <0.001 |
| **Education, N (%)** |  |  |  |
| *Less than HS* | 141 (18) | 109 (15) | <0.001 |
| *HS graduate* | 167 (21) | 92 (13) |  |
| *Some college* | 248 (32) | 220 (30) |  |
| *College grad or greater* | 221 (28) | 300 (42) |  |
| *Unknown* | 0 (0) | 1 (0.1) |  |
| **Income, N (%)** |  |  |  |
| *≤ $20,000* | 243 (31) | 150 (21) | <0.001 |
| *$20,001-50,000* | 211 (27) | 190 (26) |  |
| *$50,001-100,000* | 145 (19) | 189 (26) |  |
| *≥ $100,001* | 72 (9) | 91 (13) |  |
| *Did not answer* | 106 (14) | 102 (14) |  |
| **Insurance, N (%)** |  |  |  |
| *None* | 70 (9) | 45 (6) | <0.001 |
| *Any Public* | 456 (59) | 330 (46) |  |
| *Private* | 101 (13) | 149 (21) |  |
| *Unknown* | 150 (19) | 198 (27) |  |
| **Illicit drug use, N (%)** | 248 (32) | 257 (36) | 0.132 |
| **Current smoker, N (%)** | 70 (9) | 61 (8) | 0.701 |
| **eGFR, mL/min/1.73m^2^ (mean** ± **SD)** | 41.7 ± 18.2 | 50.8 ± 21.2 | <0.001 |
| **Urine protein-to-creatinine ratio, mg/mg (median [IQR])** | 0.19 (0.08-0.80) | 0.12 (0.06-0.46) | <0.001 |
| **Hypertension, N (%)** | 768 (99) | 626 (87) | <0.001 |
| **Number of antihypertensive medications, (median [IQR])** | 3 (3-4) | 2 (1-2) | <0.001 |
| **Diabetes, N (%)** | 450 (58) | 292 (40) | <0.001 |
| **Cardiovascular disease, N (%)** | 423 (54) | 161 (22) | <0.001 |
| **Congestive heart failure, N (%)** | 119 (15) | 27 (4) | <0.001 |
| **Atrial fibrillation, N (%)** | 201 (26) | 91 (13) | <0.001 |
| **Dyslipidemia, N (%)** | 739 (95) | 646 (89) | <0.001 |

Abbreviations: BMI, body mass index; eGFR, estimated glomerular filtration rate; HS, high school; IQR, interquartile range; SD, standard deviation.

## **Table S3. Baseline characteristics of individuals on calcium channel blockers (CCBs) vs. those not on CCBs.**

|  | **CCBs** | **No CCBs** | **p-value** |
| --- | --- | --- | --- |
| **N (%)** | 638 (43) | 861 (57) |  |
| **Age, years (mean** ± **SD)** | 64.4 ± 9.6 | 62.1 ± 10.6 | <0.001 |
| **Female Sex, N (%)** | 266 (42) | 395 (46) | 0.107 |
| **Race/Ethnicity, N (%)** |  |  |  |
| *White* | 238 (37) | 441 (51) | <0.001 |
| *Black* | 302 (47) | 279 (32) |  |
| *Hispanic* | 72 (11) | 110 (13) |  |
| *Other* | 26 (4) | 31 (4) |  |
| **BMI, kg/m^2^ (mean** ± **SD)** | 31.9 ± 6.9 | 31.2 ± 7.0 | 0.054 |
| **Education, N (%)** |  |  |  |
| *Less than HS* | 122 (19) | 128 (15) | 0.001 |
| *HS graduate* | 117 (18) | 142 (16) |  |
| *Some college* | 214 (34) | 254 (30) |  |
| *College grad or greater* | 185 (29) | 336 (39) |  |
| *Unknown* | 0 (0) | 1 (0.1) |  |
| **Income, N (%)** |  |  |  |
| *≤ $20,000* | 178 (28) | 215 (25) | 0.004 |
| *$20,001-50,000* | 189 (30) | 212 (25) |  |
| *$50,001-100,000* | 123 (19) | 211 (25) |  |
| *≥ $100,001* | 54 (8) | 109 (13) |  |
| *Did not answer* | 94 (15) | 114 (13) |  |
| **Insurance, N (%)** |  |  |  |
| *None* | 57 (9) | 58 (7) | <0.001 |
| *Any Public* | 366 (57) | 420 (49) |  |
| *Private* | 77 (12) | 173 (20) |  |
| *Unknown* | 138 (22) | 210 (25) |  |
| **Illicit drug use, N (%)** | 221 (35) | 284 (33) | 0.503 |
| **Current smoker, N (%)** | 69 (11) | 62 (7.2) | 0.014 |
| **eGFR, mL/min/1.73m^2^ (mean** ± **SD)** | 42.0 ± 19.3 | 49.1 ± 20.3 | <0.001 |
| **Urine protein-to-creatinine ratio, mg/mg (median [IQR])** | 0.26 (0.09-0.96) | 0.12 (0.06-0.40) | <0.001 |
| **Hypertension, N (%)** | 637 (99.8) | 757 (88) | <0.001 |
| **Number of antihypertensive medications, (median [IQR])** | 3 (3-4) | 2 (1-3) | <0.001 |
| **Diabetes, N (%)** | 348 (55) | 394 (46) | 0.001 |
| **Cardiovascular disease, N (%)** | 289 (45) | 295 (34) | <0.001 |
| **Congestive heart failure, N (%)** | 67 (10) | 79 (9) | 0.392 |
| **Atrial fibrillation, N (%)** | 149 (23) | 143 (17) | 0.001 |
| **Dyslipidemia, N (%)** | 604 (95) | 781 (91) | 0.004 |

Abbreviations: BMI, body mass index; CCBs, calcium channel blockers; eGFR, estimated glomerular filtration rate; HS, high school; IQR, interquartile range; SD, standard deviation.

## **Table S4. Baseline characteristics of individuals on thiazide/loop diuretics vs. those not on thiazide/loop diuretics.**

|  | **Thiazide/loop diuretics** | **No thiazide/loop diuretics** | **p-value** |
| --- | --- | --- | --- |
| **N (%)** | 756 (50) | 743 (50) |  |
| **Age, years (mean** ± **SD)** | 64.8 ± 9.2 | 61.3 ± 11.0 | <0.001 |
| **Female Sex, N (%)** | 333 (44) | 328 (44) | 0.970 |
| **Race/Ethnicity, N (%)** |  |  |  |
| *White* | 284 (38) | 395 (53) | <0.001 |
| *Black* | 343 (45) | 238 (32) |  |
| *Hispanic* | 103 (14) | 79 (11) |  |
| *Other* | 26 (3) | 31 (4) |  |
| **BMI, kg/m^2^ (mean** ± **SD)** | 32.3 ± 6.9 | 29.6 ± 6.0 | <0.001 |
| **Education, N (%)** |  |  |  |
| *Less than HS* | 150 (20) | 100 (13) | <0.001 |
| *HS graduate* | 138 (18) | 121 (16) |  |
| *Some college* | 249 (33) | 219 (29) |  |
| *College grad or greater* | 219 (29) | 302 (41) |  |
| *Unknown* | 0 (0) | 1 (0.1) |  |
| **Income, N (%)** |  |  |  |
| *≤ $20,000* | 233 (31) | 160 (22) | <0.001 |
| *$20,001-50,000* | 207 (27) | 194 (26) |  |
| *$50,001-100,000* | 139 (18) | 195 (26) |  |
| *≥ $100,001* | 67 (9) | 96 (13) |  |
| *Did not answer* | 110 (15) | 98 (13) |  |
| **Insurance, N (%)** |  |  |  |
| *None* | 65 (9) | 50 (7) | <0.001 |
| *Any Public* | 447 (59) | 339 (46) |  |
| *Private* | 100 (13) | 150 (20) |  |
| *Unknown* | 144 (19) | 204 (27) |  |
| **Illicit drug use, N (%)** | 241 (32) | 264 (36) | 0.135 |
| **Current smoker, N (%)** | 67 (9) | 64 (9) | 0.865 |
| **eGFR, mL/min/1.73m^2^ (mean** ± **SD)** | 40.3 ± 17.7 | 51.9 ± 21.0 | <0.001 |
| **Urine protein-to-creatinine ratio, mg/mg (median [IQR])** | 0.18 (0.08-0.76) | 0.13 (0.06-0.49) | <0.001 |
| **Hypertension, N (%)** | 750 (99) | 644 (87) | <0.001 |
| **Number of antihypertensive medications, (median [IQR])** | 3 (3-4) | 2 (1-2) | <0.001 |
| **Diabetes, N (%)** | 472 (62) | 270 (36) | <0.001 |
| **Cardiovascular disease, N (%)** | 362 (48) | 222 (30) | <0.001 |
| **Congestive heart failure, N (%)** | 115 (15) | 31 (4) | <0.001 |
| **Atrial fibrillation, N (%)** | 190 (25) | 102 (14) | <0.001 |
| **Dyslipidemia, N (%)** | 725 (96) | 660 (89) | <0.001 |

Abbreviations: BMI, body mass index; eGFR, estimated glomerular filtration rate; HS, high school; IQR, interquartile range; SD, standard deviation.

## **Table S5. Sensitivity analysis: Baseline characteristics for additional medication classes.**

|  | **Overall** | **Thiazide diuretics** | **Loop diuretics** | **Alphablockers** | **Potassium-sparing diuretics** |
| --- | --- | --- | --- | --- | --- |
| **N (%)** | 1 499 | 315 (21) | 524 (35) | 301 (20) | 126 (8) |
| **Age, years (mean** ± **SD)** | 63.1 ± 10.3 | 64.8 ± 9.1* | 64.8 ± 9.3* | 66.0 ± 8.7* | 64.1 ± 9.4 |
| **Female Sex, N (%)** | 661 (44.1) | 149 (47) | 223 (43) | 71 (24)* | 57 (45) |
| **Race/Ethnicity, N (%)** |  | * | * |  | * |
| *White* | 679 (45.3) | 133 (42) | 183 (35) | 123 (41) | 56 (44) |
| *Black* | 581 (38.8) | 143 (45) | 244 (47) | 125 (42) | 64 (51) |
| *Hispanic* | 182 (12.1) | 27 (9) | 82 (16) | 43 (14) | 5 (4) |
| *Other* | 57 (3.8) | 12 (4) | 15 (3) | 10 (3) | 1 (1) |
| **BMI, kg/m^2^ (mean** ± **SD)** | 31.5 ± 7.0 | 32.3 ± 6.9* | 34.0 ± 7.5* | 32.0 ± 5.7 | 32.5 ± 6.8 |
| **Education, N (%)** |  |  | * | * |  |
| *Less than HS* | 250 (16.7) | 46 (15) | 118 (23) | 64 (21) | 15 (12) |
| *HS graduate* | 259 (17.3) | 58 (18) | 96 (18) | 57 (19) | 18 (14) |
| *Some college* | 468 (31.2) | 98 (31) | 181 (35) | 98 (33) | 47 (37) |
| *College grad or greater* | 521 (34.8) | 113 (36) | 129 (25) | 82 (27) | 46 (37) |
| *Unknown* | 1 (0.1) | 0 (0) | 0 (0) | 0 (0) | 0 (0) |
| **Income, N (%)** |  |  | * | * |  |
| *≤ $20,000* | 393 (26.2) | 80 (25) | 174 (33) | 101 (34) | 28 (22) |
| *$20,001-50,000* | 401 (26.8) | 85 (27) | 150 (29) | 80 (27) | 38 (31) |
| *$50,001-100,000* | 334 (22.3) | 68 (22) | 82 (16) | 53 (18) | 26 (21) |
| *≥ $100,001* | 163 (10.9) | 36 (11) | 42 (8) | 31 (10) | 18 (14) |
| *Did not answer* | 208 (13.9) | 46 (15) | 76 (14) | 36 (12) | 16 (13) |
| **Insurance, N (%)** |  |  | * | * | * |
| *None* | 115 (7.7) | 20 (6) | 51 (10) | 18 (6) | 4 (3) |
| *Any Public* | 786 (52.4) | 177 (56) | 314 (60) | 207 (69) | 63 (50) |
| *Private* | 250 (16.7) | 48 (15) | 62 (12) | 26 (9) | 19 (15) |
| *Unknown* | 348 (23.2) | 70 (22) | 97 (19) | 50 (17) | 40 (32) |
| **Illicit drug use, N (%)** | 505 (33.7) | 109 (35) | 163 (31) | 96 (32) | 48 (38) |
| **Current smoker, N (%)** | 131 (8.7) | 31 (10) | 44 (8) | 23 (8) | 13 (10) |
| **eGFR, mL/min/1.73m^2^ (mean** ± **SD)** | 46.1 ± 20.2 | 48.1 ± 17.3* | 36.1 ± 16.4* | 39.8 ± 18.8* | 47.8 ± 18.1 |
| **Urine protein-to-creatinine ratio, mg/mg (median [IQR])** | 0.15 (0.07-0.60) | 0.10 (0.06-0.32)* | 0.25 (0.10-1.00)* | 0.27 (0.09-1.08)* | 0.10 (0.06-0.24)* |
| **Hypertension, N (%)** | 1 394 (93.0) | 311 (99)* | 521 (99)* | 298 (99)* | 123 (98)* |
| **Number of antihypertensive medications, (median [IQR])** | 3 (1-4) | 3 (2-4)* | 4 (3-4)* | 4 (3-5)* | 3 (2-4)* |
| **Diabetes, N (%)** | 742 (49.5) | 167 (53) | 355 (68)* | 192 (64)* | 60 (48) |
| **Cardiovascular disease, N (%)** | 584 (39.0) | 119 (38) | 281 (54)* | 171 (57)* | 62 (49)* |
| **Congestive heart failure, N (%)** | 146 (9.7) | 22 (7) | 104 (20)* | 58 (19)* | 24 (19)* |
| **Atrial fibrillation, N (%)** | 292 (19.5) | 70 (22) | 143 (27)* | 78 (26)* | 33 (26)* |
| **Dyslipidemia, N (%)** | 1 385 (92.4) | 296 (94) | 511 (98)* | 294 (98)* | 121 (96) |

Abbreviations: BMI, body mass index; eGFR, estimated glomerular filtration rate; HS, high school; IQR, interquartile range; SD, standard deviation.

*Indicates that there is a significant difference for this baseline characteristic comparing the group of individuals on the medication class (displayed in the table) vs. those not on the medication class (not displayed on the table, in the supplementary appendix Tables S8-11).

Baseline characteristics that are continuous variables, with the exception of urine protein-to-creatinine ratio (i.e. age, BMI, eGFR, hemoglobin, and number of antihypertensive medications) were compared using a t-test of means with a two-sided alpha significance of 0.05. Urine protein-to-creatinine ratio and number of antihypertensive medications was compared with a Kruskal-Wallis equality of proportions rank test with a two-sided alpha significance of 0.05.

The proportion of people on each medication class versus not with each categorical and binary baseline characteristic (sex, race/ethnicity, education, income, insurance, illicit drug use, current smoker, hypertension, diabetes, cardiovascular disease, heart failure, atrial fibrillation, and high cholesterol) were compared using a chi-squared test with a significance level of 0.05.

## **Table S6. Sensitivity analysis: Baseline characteristics of individuals on thiazide diuretics vs. those not on thiazide diuretics.**

|  | **Thiazide diuretics** | **No thiazide diuretics** | **p-value** |
| --- | --- | --- | --- |
| **N (%)** | 315 (21) | 1 184 (79) |  |
| **Age, years (mean** ± **SD)** | 64.8 ± 9.1 | 62.6 ± 10.5 | <0.001 |
| **Female Sex, N (%)** | 149 (47) | 512 (43) | 0.197 |
| **Race/Ethnicity, N (%)** |  |  |  |
| *White* | 133 (42) | 546 (46) | 0.023 |
| *Black* | 143 (45) | 438 (37) |  |
| *Hispanic* | 27 (9) | 155 (13) |  |
| *Other* | 12 (4) | 45 (4) |  |
| **BMI, kg/m^2^ (mean** ± **SD)** | 32.3 ± 6.9 | 31.3 ± 7.0 | 0.019 |
| **Education, N (%)** |  |  |  |
| *Less than HS* | 46 (15) | 204 (17) | 0.784 |
| *HS graduate* | 58 (18) | 201 (17) |  |
| *Some college* | 98 (31) | 370 (31) |  |
| *College grad or greater* | 113 (36) | 408 (34) |  |
| *Unknown* | 0 (0) | 1 (0.1) |  |
| **Income, N (%)** |  |  |  |
| *≤ $20,000* | 80 (25) | 313 (26) | 0.977 |
| *$20,001-50,000* | 85 (27) | 316 (27) |  |
| *$50,001-100,000* | 68 (22) | 266 (22) |  |
| *≥ $100,001* | 36 (11) | 127 (11) |  |
| *Did not answer* | 46 (15) | 162 (14) |  |
| **Insurance, N (%)** |  |  |  |
| *None* | 20 (6) | 95 (8) | 0.449 |
| *Any Public* | 177 (56) | 609 (51) |  |
| *Private* | 48 (15) | 202 (17) |  |
| *Unknown* | 70 (22) | 278 (23) |  |
| **Illicit drug use, N (%)** | 109 (35) | 396 (33) | 0.699 |
| **Current smoker, N (%)** | 31 (10) | 100 (8) | 0.436 |
| **eGFR, mL/min/1.73m^2^ (mean** ± **SD)** | 48.1 ± 17.3 | 45.5 ± 20.9 | 0.049 |
| **Urine protein-to-creatinine ratio, mg/mg (median [IQR])** | 0.10 (0.06-0.32) | 0.18 (0.07-0.67) | <0.001 |
| **Hypertension, N (%)** | 311 (99) | 1 083 (91) | <0.001 |
| **Number of antihypertensive medications, (median [IQR])** | 3 (2-4) | 2 (1-3) | <0.001 |
| **Diabetes, N (%)** | 167 (53) | 575 (49) | 0.160 |
| **Cardiovascular disease, N (%)** | 119 (38) | 465 (39) | 0.628 |
| **Congestive heart failure, N (%)** | 22 (7) | 124 (10) | 0.063 |
| **Atrial fibrillation, N (%)** | 70 (22) | 222 (19) | 0.167 |
| **Dyslipidemia, N (%)** | 296 (94) | 1 089 (92) | 0.236 |

Abbreviations: BMI, body mass index; eGFR, estimated glomerular filtration rate; HS, high school; IQR, interquartile range; SD, standard deviation.

## **Table S7. Sensitivity analysis: Baseline characteristics of individuals on loop diuretics vs. those not on loop diuretics.**

|  | **Loop diuretics** | **No loop diuretics** | **p-value** |
| --- | --- | --- | --- |
| **N (%)** | 524 (35) | 975 (65) |  |
| **Age, years (mean** ± **SD)** | 64.8 ± 9.3 | 62.1 ± 10.6 | <0.001 |
| **Female Sex, N (%)** | 223 (43) | 438 (45) | 0.379 |
| **Race/Ethnicity, N (%)** |  |  |  |
| *White* | 183 (35) | 496 (51) | <0.001 |
| *Black* | 244 (47) | 337 (35) |  |
| *Hispanic* | 82 (16) | 100 (10) |  |
| *Other* | 15 (3) | 42 (4) |  |
| **BMI, kg/m^2^ (mean** ± **SD)** | 34.0 ± 7.5 | 30.2 ± 6.3 | <0.001 |
| **Education, N (%)** |  |  |  |
| *Less than HS* | 118 (23) | 132 (14) | <0.001 |
| *HS graduate* | 96 (18) | 163 (17) |  |
| *Some college* | 181 (35) | 287 (29) |  |
| *College grad or greater* | 129 (25) | 392 (40) |  |
| *Unknown* | 0 (0) | 1 (0.1) |  |
| **Income, N (%)** |  |  |  |
| *≤ $20,000* | 174 (33) | 219 (22) | <0.001 |
| *$20,001-50,000* | 150 (29) | 251 (26) |  |
| *$50,001-100,000* | 82 (16) | 252 (26) |  |
| *≥ $100,001* | 42 (8) | 121 (12) |  |
| *Did not answer* | 76 (14) | 132 (14) |  |
| **Insurance, N (%)** |  |  |  |
| *None* | 51 (10) | 64 (7) | <0.001 |
| *Any Public* | 314 (60) | 472 (48) |  |
| *Private* | 62 (12) | 188 (19) |  |
| *Unknown* | 97 (19) | 251 (26) |  |
| **Illicit drug use, N (%)** | 163 (31) | 342 (35) | 0.121 |
| **Current smoker, N (%)** | 44 (8) | 87 (8) | 0.731 |
| **eGFR, mL/min/1.73m^2^ (mean** ± **SD)** | 36.1 ± 16.4 | 51.4 ± 20.0 | <0.001 |
| **Urine protein-to-creatinine ratio, mg/mg (median [IQR])** | 0.25 (0.10-1.00) | 0.12 (0.06-0.44) | <0.001 |
| **Hypertension, N (%)** | 521 (99) | 873 (90) | <0.001 |
| **Number of antihypertensive medications, (median [IQR])** | 4 (3-4) | 2 (1-3) | <0.001 |
| **Diabetes, N (%)** | 355 (68) | 387 (40) | <0.001 |
| **Cardiovascular disease, N (%)** | 281 (54) | 303 (31) | <0.001 |
| **Congestive heart failure, N (%)** | 104 (20) | 42 (4) | <0.001 |
| **Atrial fibrillation, N (%)** | 143 (27) | 149 (15) | <0.001 |
| **Dyslipidemia, N (%)** | 511 (98) | 874 (90) | <0.001 |

Abbreviations: BMI, body mass index; eGFR, estimated glomerular filtration rate; HS, high school; IQR, interquartile range; SD, standard deviation.

## **Table S8. Sensitivity analysis: Baseline characteristics of individuals on alpha blockers vs. those not on alpha blockers.**

|  | **Alpha blockers** | **No alpha blockers** | **p-value** |
| --- | --- | --- | --- |
| **N (%)** | 301 (20) | 1 198 (80) |  |
| **Age, years (mean** ± **SD)** | 66.0 ± 8.7 | 62.3 ± 10.5 | <0.001 |
| **Female Sex, N (%)** | 71 (24) | 590 (49) | <0.001 |
| **Race/Ethnicity, N (%)** |  |  |  |
| *White* | 123 (41) | 556 (46) | 0.258 |
| *Black* | 125 (42) | 456 (38) |  |
| *Hispanic* | 43 (14) | 139 (12) |  |
| *Other* | 10 (3) | 47 (4) |  |
| **BMI, kg/m^2^ (mean** ± **SD)** | 32.0 ± 5.7 | 31.4 ± 7.2 | 0.141 |
| **Education, N (%)** |  |  |  |
| *Less than HS* | 64 (21) | 186 (16) | 0.018 |
| *HS graduate* | 57 (19) | 202 (17) |  |
| *Some college* | 98 (33) | 370 (31) |  |
| *College grad or greater* | 82 (27) | 439 (37) |  |
| *Unknown* | 0 (0) | 1 (0.1) |  |
| **Income, N (%)** |  |  |  |
| *≤ $20,000* | 101 (34) | 292 (24) | 0.014 |
| *$20,001-50,000* | 80 (27) | 321 (27) |  |
| *$50,001-100,000* | 53 (18) | 281 (23) |  |
| *≥ $100,001* | 31 (10) | 132 (11) |  |
| *Did not answer* | 36 (12) | 172 (14) |  |
| **Insurance, N (%)** |  |  |  |
| *None* | 18 (6) | 97 (8) | <0.001 |
| *Any Public* | 207 (69) | 579 (48) |  |
| *Private* | 26 (9) | 224 (19) |  |
| *Unknown* | 50 (17) | 298 (25) |  |
| **Illicit drug use, N (%)** | 96 (32) | 409 (34) | 0.461 |
| **Current smoker, N (%)** | 23 (8) | 108 (9) | 0.451 |
| **eGFR, mL/min/1.73m^2^ (mean** ± **SD)** | 39.8 ± 18.8 | 47.7 ± 20.2 | <0.001 |
| **Urine protein-to-creatinine ratio, mg/mg (median [IQR])** | 0.27 (0.09-1.08) | 0.14 (0.07-0.51) | <0.001 |
| **Hypertension, N (%)** | 298 (99) | 1 096 (91) | <0.001 |
| **Number of antihypertensive medications, (median [IQR])** | 4 (3-5) | 2 (1-3) | <0.001 |
| **Diabetes, N (%)** | 192 (64) | 550 (46) | <0.001 |
| **Cardiovascular disease, N (%)** | 171 (57) | 413 (34) | <0.001 |
| **Congestive heart failure, N (%)** | 58 (19) | 88 (7) | <0.001 |
| **Atrial fibrillation, N (%)** | 78 (26) | 214 (18) | 0.002 |
| **Dyslipidemia, N (%)** | 294 (98) | 1 091 (91) | <0.001 |

Abbreviations: BMI, body mass index; eGFR, estimated glomerular filtration rate; HS, high school; IQR, interquartile range; SD, standard deviation.

## **Table S9. Sensitivity analysis: Baseline characteristics of individuals on potassium-sparing diuretics vs. those not on potassium-sparing diuretics.**

|  | **Potassium-sparing diuretics** | **No potassium-sparing diuretic** | **p-value** |
| --- | --- | --- | --- |
| **N (%)** | 126 (8) | 1 373 (92) |  |
| **Age, years (mean** ± **SD)** | 64.1 ± 9.4 | 63.0 ± 10.3 | 0.218 |
| **Female Sex, N (%)** | 57 (45) | 604 (44) | 0.787 |
| **Race/Ethnicity, N (%)** |  |  |  |
| *White* | 56 (44) | 623 (45) | 0.001 |
| *Black* | 64 (51) | 517 (38) |  |
| *Hispanic* | 5 (4) | 177 (13) |  |
| *Other* | 1 (1) | 56 (4) |  |
| **BMI, kg/m^2^ (mean** ± **SD)** | 32.5 ± 6.8 | 31.4 ± 7.0 | 0.094 |
| **Education, N (%)** |  |  |  |
| *Less than HS* | 15 (12) | 235 (17) | 0.350 |
| *HS graduate* | 18 (14) | 241 (18) |  |
| *Some college* | 47 (37) | 421 (31) |  |
| *College grad or greater* | 46 (37) | 475 (35) |  |
| *Unknown* | 0 (0) | 1 (0.1) |  |
| **Income, N (%)** |  |  |  |
| *≤ $20,000* | 28 (22) | 365 (27) | 0.523 |
| *$20,001-50,000* | 38 (31) | 363 (31) |  |
| *$50,001-100,000* | 26 (21) | 26 (21) |  |
| *≥ $100,001* | 18 (14) | 18 (14) |  |
| *Did not answer* | 16 (13) | 16 (13) |  |
| **Insurance, N (%)** |  |  |  |
| *None* | 4 (3) | 111 (8) | 0.040 |
| *Any Public* | 63 (50) | 723 (53) |  |
| *Private* | 19 (15) | 231 (17) |  |
| *Unknown* | 40 (32) | 308 (22) |  |
| **Illicit drug use, N (%)** | 48 (38) | 457 (33) | 0.274 |
| **Current smoker, N (%)** | 13 (10) | 118 (9) | 0.512 |
| **eGFR, mL/min/1.73m^2^ (mean** ± **SD)** | 47.8 ± 18.1 | 45.9 ± 20.4 | 0.308 |
| **Urine protein-to-creatinine ratio, mg/mg (median [IQR])** | 0.10 (0.06-0.24) | 0.16 (0.06-0.63) | 0.002 |
| **Hypertension, N (%)** | 123 (98) | 1 271 (93) | 0.034 |
| **Number of antihypertensive medications, (median [IQR])** | 3 (2-4) | 2 (1-4) | <0.001 |
| **Diabetes, N (%)** | 60 (48) | 682 (50) | 0.659 |
| **Cardiovascular disease, N (%)** | 62 (49) | 522 (38) | 0.014 |
| **Congestive heart failure, N (%)** | 24 (19) | 122 (9) | <0.001 |
| **Atrial fibrillation, N (%)** | 33 (26) | 259 (19) | 0.047 |
| **Dyslipidemia, N (%)** | 121 (96) | 1 264 (92) | 0.108 |

Abbreviations: BMI, body mass index; eGFR, estimated glomerular filtration rate; HS, high school; IQR, interquartile range; SD, standard deviation.

## **Table S10. Baseline office and 24-hour ambulatory blood pressure among those using and not using each medication class.**

| Mean, mmHg ± SD | **RASis** | **Not on RASis** | **Beta Blockers** | **Not on beta blockers** | **CCBs** | **Not on CCBs** | **Thiazide or loop diuretics** | **Not on thiazide or loop diuretics** |
| --- | --- | --- | --- | --- | --- | --- | --- | --- |
| N | 984 | 515 | 777 | 722 | 638 | 861 | 756 | 743 |
| Office SBP | 125.5 ±19.6 | 127.5 ±21.7 | 128.4 ±21.2 | 123.8 ±19.1 | 129.3 ±19.2 | 123.9 ±20.8 | 128.4 ±20.8 | 124.0±19.5 |
| p-value | 0.073 | | <0.001 | | <0.001 | | <0.001 | |
| Office DBP | 68.5 ±12.1 | 70.8 ±12.1 | 68.5 ±12.7 | 70.0 ±11.5 | 68.7 ±12.3 | 69.7 ±12.0 | 67.7 ±12.7 | 70.8 ±11.3 |
| p-value | <0.001 | | 0.015 | | 0.110 | | <0.001 | |
| 24-hour SBP Average | 127.7 ±15.4 | 129.7 ±17.0 | 130.4 ±16.3 | 126.1 ±15.3 | 131.7 ±14.9 | 125.9 ±16.2 | 130.1 ±15.9 | 126.6±15.8 |
| p-value | 0.021 | | <0.001 | | <0.001 | | <0.001 | |
| 24-hour DBP Average | 71.6 ±9.4 | 74.0 ±9.8 | 71.9 ±10.0 | 73.1 ±9.1 | 72.8 ±9.5 | 72.2 ±9.7 | 71.2 ±9.9 | 73.7 ±9.2 |
| p-value | <0.001 | | 0.016 | | 0.234 | | <0.001 | |
| Daytime SBP Average | 131.5 ±15.2 | 133.6 ±16.5 | 133.6 ±16.2 | 130.8 ±15.1 | 134.9 ±14.5 | 130.3 ±16.3 | 133.5 ±15.7 | 131.0±15.6 |
| p-value | 0.014 | | 0.001 | | <0.001 | | 0.002 | |
| Daytime DBP Average | 74.8 ±9.8 | 77.5 ±10.2 | 74.7 ±10.3 | 76.8 ±9.7 | 75.6 ±9.7 | 75.7 ±10.3 | 74.0 ±10.2 | 77.4 ±9.6 |
| p-value | <0.001 | | <0.001 | | 0.821 | | <0.001 | |
| Nighttime SBP Average | 120.2 ±18.3 | 122.3 ±20.5 | 124.1 ±19.6 | 117.4 ±17.9 | 125.3 ±18.5 | 117.6 ±18.9 | 123.5 ±19.1 | 118.3±18.8 |
| p-value | 0.039 | | <0.001 | | <0.001 | | <0.001 | |
| Nighttime DBP Average | 65.9 ±10.6 | 67.8 ±11.4 | 66.7 ±11.6 | 66.5 ±10.1 | 67.7 ±11.0 | 65.7 ±10.8 | 65.9 ±11.3 | 67.2 ±10.4 |
| p-value | 0.002 | | 0.721 | | 0.001 | | 0.021 | |

Abbreviations: CCBs, calcium channel blockers; DBP, diastolic blood pressure; RASis, renin angiotensin system inhibitors; mmHg, millimetres of mercury; SBP, systolic blood pressure; SD, standard deviation.

Blood pressure characteristics at baseline office visit and 24-hour ambulatory blood pressure are compared between the group of individuals on the medication class vs. those not on the medication class, using a two-sample t-test with a two-sided alpha of 0.05. Note this is the same table as Table 3 in the manuscript but also includes mean values for those not on each medication class and p-values.

## **Table S11. Sensitivity analysis: Baseline office and 24-hour ambulatory blood pressure of those using and not using each additional medication classes.**

| Mean, mmHg ± SD | **Thiazide diuretics** | **Not on thiazide diuretics** | **Loop diuretics** | **Not on loop diuretics** | **Alpha blockers** | **Not on alpha blockers** | **Potassium-sparing diuretics** | **Not on potassium-sparing diuretics** |
| --- | --- | --- | --- | --- | --- | --- | --- | --- |
| N | 315 | 1 184 | 524 | 975 | 301 | 1 198 | 126 | 1 373 |
| Office SBP | 126.5 ±20.3 | 126.1 ±20.3 | 129.1 ±21.1 | 124.6 ±19.7 | 127.9 ±22.0 | 125.8 ±19.9 | 121.9 ±17.5 | 126.6 ±20.5 |
| p-value | 0.754 | | <0.001 | | 0.108 | | 0.014 | |
| Office DBP | 69.2 ±12.5 | 69.3 ±12.1 | 66.8 ±12.6 | 70.6 ±11.7 | 67.1 ±13.1 | 69.8 ±11.8 | 68.2 ±13.1 | 69.3 ±12.1 |
| p-value | 0.900 | | <0.001 | | <0.001 | | 0.305 | |
| 24-hour SBP Average | 128.0±15.7 | 128.5±16.0 | 131.3 ±16.1 | 126.8 ±15.7 | 133.2 ±16.1 | 127.1 ±15.7 | 125.5 ±14.8 | 128.6 ±16.0 |
| p-value | 0.661 | | <0.001 | | <0.001 | | 0.033 | |
| 24-hour DBP Average | 71.4±9.7 | 72.7±9.6 | 70.9 ±9.8 | 73.2 ±9.4 | 72.8 ±10.0 | 72.3 ±9.5 | 70.9 ±10.3 | 72.6 ±9.5 |
| p-value | 0.033 | | <0.001 | | 0.417 | | 0.065 | |
| Daytime SBP Average | 132.1±16.0 | 132.3±15.6 | 134.4 ±15.6 | 131.1 ±15.7 | 135.5 ±15.9 | 131.4 ±15.6 | 129.0 ±14.9 | 132.6 ±15.8 |
| p-value | 0.832 | | <0.001 | | <0.001 | | 0.014 | |
| Daytime DBP Average | 74.8±10.2 | 75.9±10.0 | 73.5 ±10.0 | 76.9 ±9.9 | 75.2 ±10.4 | 75.8 ±10.0 | 74.1 ±10.9 | 75.8 ±10.0 |
| p-value | 0.070 | | <0.001 | | 0.343 | | 0.063 | |
| Nighttime SBP Average | 120.4±18.2 | 121.0±19.3 | 125.3 ±19.4 | 118.5 ±18.5 | 128.5 ±19.5 | 119.0 ±18.5 | 118.7 ±16.8 | 121.1 ±19.3 |
| p-value | 0.611 | | <0.001 | | <0.001 | | 0.177 | |
| Nighttime DBP Average | 65.4±11.0 | 66.9±10.9 | 66.2 ±11.3 | 66.8 ±10.7 | 68.5 ±11.3 | 66.1 ±10.8 | 65.4 ±11.1 | 66.7 ±10.9 |
| p-value | 0.038 | | 0.295 | | 0.001 | | 0.191 | |

Abbreviations: DBP, diastolic blood pressure; mmHg, millimetres of mercury; SBP, systolic blood pressure; SD, standard deviation.

*Blood pressure characteristics at baseline office visit and 24-hour ambulatory blood pressure are compared between the group of individuals on the medication class vs. those not on the medication class, for medication classes included in sensitivity analysis, using a two-sample t-test with a two-sided alpha of 0.05.

## **Table S12. P-values for ambulatory blood pressure phenotype by medication class: (Table 4)**

|  | **RASis** | **Beta blockers** | **CCBs** | **Thiazide or loop diuretics** |
| --- | --- | --- | --- | --- |
| Controlled hypertension | REFERENCE | | | |
| White coat effect | 0.706 | 0.175 | 0.241 | 0.553 |
| Sustained hypertension | 0.003* | 0.369 | 0.454 | 0.722 |
| Masked uncontrolled hypertension | 0.124 | 0.006* | 0.015 | 0.112 |
| Acronyms: CCBs, calcium channel blockers; RASis, renin angiotensin system inhibitors. This table indicates the results of the adjusted multivariate logistic regression of ambulatory blood pressure phenotype by medication class. *Denotes significance using a Bonferroni corrected alpha of 0.0125. | | | | |

## **Table S13. Sensitivity analysis: Ambulatory blood pressure phenotype by medication class, including additional medication classes.**

|  | **RASis** | **Beta blockers** | **CCBs** | **Thiazide diuretics** | **Loop diuretics** | **Alpha blockers** | **K+ sparing diuretics** |
| --- | --- | --- | --- | --- | --- | --- | --- |
| Controlled hypertension | REFERENCE | | | | | | |
| White coat effect,  OR (95% CI) | 0.85 (0.47, 1.55) p=0.600 | 1.63 (0.90, 2.94) p=0.105 | 1.43 (0.81, 2.51) p=0.215 | 0.94 (0.49, 1.81) p=0.864 | 0.48* (0.24, 0.95) p=0.035 | 0.63 (0.27, 1.46) p=0.284 | 1.57 (0.59, 4.18) p=0.369 |
| Sustained hypertension, OR (95% CI) | 0.59* (0.42, 0.84) p=0.003 | 1.17 (0.82, 1.68) p=0.386 | 1.14 (0.82, 1.60) p=0.438 | 0.97 (0.64, 1.49) p=0.905 | 0.90 (0.60, 1.35) p=0.626 | 1.09 (0.71, 1.68) p=0.693 | 0.60 (0.30, 1.20) p=0.146 |
| Masked uncontrolled hypertension, OR (95% CI) | 0.81 (0.61, 1.08) p=0.145 | 1.42* (1.07, 1.89) p=0.017 | 1.40* (1.07, 1.83) p=0.015 | 0.80 (0.57, 1.12) p=0.196 | 0.86 (0.62, 1.19) p=0.373 | 1.28 (0.91, 1.80) p=0.159 | 0.79 (0.48, 1.29) p=0.343 |
| Acronyms: CCBs, calcium channel blockers; CI, confidence interval; OR, odds ratio; RASis, renin angiotensin system inhibitors. This table demonstrates the results of separate adjusted nominal logistic regression models for each ABPM phenotype to determine if there is an independent association between each medication class and that specific ABPM phenotypes.  *Denotes statistically significant values, using a two-sided alpha significance level of 0.05. | | | | | | | |

## **Table S14. P-values for nocturnal blood pressure and blood pressure variability analyses (secondary outcomes, Table 4)**

|  | **RASis** | **Beta blockers** | **CCBs** | **Thiazide/loop diuretics** |
| --- | --- | --- | --- | --- |
| **Nocturnal blood pressure** | | | | |
| Nocturnal hypertension (SBP≥120 mmHg and/or DBP≥70 mmHg) | 0.008* | 0.024 | 0.011* | 0.618 |
| Non-dipping | 0.910 | 0.028* | 0.042* | 0.477 |
| **Blood pressure variability** | | | | |
| 24-hour systolic variability | 0.798 | 0.418 | 0.007* | 0.628 |
| 24-hour diastolic variability | 0.993 | 0.241 | <0.001* | 0.646 |
| Acronyms: CCBs, calcium channel blockers; mmHg, millimetres of mercury; RASis, renin angiotensin system inhibitors; SBP, systolic blood pressure.  This table indicates the adjusted multivariate logistic regression of nocturnal hypertension & non-dipping status, and adjusted multivariate linear regression of 24-hour systolic variability & 24-hour diastolic variability.  *Denotes statistically significant values, using a two-sided alpha significance level of 0.05. | | | | |

## **Table S15. Sensitivity analysis: Nocturnal blood pressure and blood pressure variability (secondary outcomes) by medication class, including additional medication classes.**

|  | **RASis** | **Beta blockers** | **CCBs** | **Thiazide diuretics** | **Loop diuretics** | **Alpha blockers** | **Potassium-sparing diuretics** |
| --- | --- | --- | --- | --- | --- | --- | --- |
| **Nocturnal blood pressure** | | | | | | | |
| Nocturnal HTN  (SBP≥120 mmHg and/or DBP≥70 mmHg) OR (95% CI) | 0.72* (0.56, 0.93) p=0.012 | 1.24 (0.96, 1.59) p=0.103 | 1.36* (1.07, 1.73) p=0.012 | 0.97 (0.72, 1.30) p=0.840 | 0.92 (0.69, 1.23) p=0.558 | 1.61* (1.17, 2.22) p=0.004 | 0.91 (0.59, 1.40) p=0.672 |
| Non-dipping OR (95% CI) | 1.04 (0.82, 1.32) p=0.722 | 1.20 (0.95, 1.53) p=0.129 | 1.26* (1.00, 1.58) p=0.045 | 1.00 (0.76, 1.33) p=0.978 | 1.22 (0.93, 1.60) p=0.157 | 1.48* (1.10, 2.01) p=0.010 | 0.98 (0.65, 1.49) p=0.937 |
| **Blood pressure variability** | | | | | | | |
| 24-hour systolic variability β coefficient (95% CI) | 0.02 (-0.24, 0.28) p=0.875 | -0.09 (-0.35, 0.17) p=0.499 | -0.35* (-0.60, -0.10) p=0.005 | 0.37* (0.06, 0.67) p=0.017 | -0.27 (-0.56, 0.03) p=0.076 | 0.04 (-0.28, 0.35) p=0.823 | -0.13 (-0.58, 0.32) p=0.564 |
| 24-hour diastolic variability  β coefficient (95% CI) | -0.02 (-0.21, 0.16) p=0.790 | -0.05 (-0.24, 0.14) p=0.600 | -0.34* (-0.52, -0.16) p<0.001 | 0.23* (0.02, 0.45) p=0.036 | -0.16 (-0.37, 0.05) p=0.138 | -0.27* (-0.49,-0.04) p=0.020 | 0.00 (-0.32,0.32) p=0.990 |
| Acronyms: β, beta coefficient; CI, confidence interval; CCBs, calcium channel blockers; mmHg, millimetres of mercury; OR, odds ratio; RASis, renin angiotensin system inhibitors; SBP, systolic blood pressure.  This table demonstrates the results of separate adjusted logistic regression models for nocturnal hypertension and non-dipping status, in addition to separate adjusted linear regression models for 24-hour systolic and diastolic variability, to determine if medication class is associated with each of these secondary outcomes.  *Denotes statistically significant values, using a two-sided alpha significance level of 0.05. | | | | | | | |

## **Table S16. Sensitivity analysis: Primary and secondary outcome analysis, using an alternative threshold for ambulatory blood pressure validity.**

|  | **RASis (N= 942)** | | **Beta blockers (N=736)** | **CCBs (N=608)** | | **Thiazide/loop diuretics (N=718)** | |
| --- | --- | --- | --- | --- | --- | --- | --- |
| **Ambulatory blood pressure phenotype** | | | | | | | |
| Controlled hypertension | | REFERENCE | | | | | |
| White coat effect | | 0.94 (0.51, 1.73) p=0.84 | 1.43 (0.79, 2.58) p=0.23 | | 1.44 (0.81, 2.55) p=0.22 | | 0.8 (0.44, 1.48) p=0.48 |
| Sustained hypertension | | 0.65 (0.45, 0.92) p=0.02* | 1.23 (0.86, 1.75) p=0.26 | | 1.09 (0.77, 1.53) p=0.62 | | 0.92 (0.63, 1.33) p=0.66 |
| Masked uncontrolled hypertension | | 0.82 (0.61, 1.1) p=0.19 | 1.5 (1.12, 2.0) p<0.01* | | 1.37 (1.04, 1.81) p=0.02* | | 0.75 (0.56, 1.01) p=0.06 |
| **Nocturnal blood pressure** | | | | | | | |
| Nocturnal HTN  (SBP≥120 mmHg and/or DBP≥70 mmHg)  OR (95% CI) | 0.71 (0.55, 0.92) p<0.01* | | 1.38 (1.07, 1.77) p=0.01* | 1.33 (1.04, 1.7) p=0.02* | | 0.90 (0.69, 1.17) p=0.43 | |
| Non-dipping OR (95% CI) | 1.02 (0.80, 1.30) p=0.85 | | 1.21 (0.95, 1.55) p=0.12 | 1.31 (1.04, 1.66) p=0.02* | | 1.08 (0.84, 1.38) p=0.55 | |
| **Blood pressure variability** | | | | | | | |
| 24-hour systolic variability β coefficient (95% CI) | 0.03 (-0.23, 0.29)  p=0.83 | | -0.04 (-0.30, 0.22)  p=0.76 | -0.39 (-0.64, -0.13)  p<0.01* | | 0.05 (-0.22, 0.32)  p=0.71 | |
| 24-hour diastolic variability  β coefficient (95% CI) | -0.01 (-0.20, 0.18)  p=0.92 | | -0.09 (-0.28, 0.10)  p=0.34 | -0.36 (-0.54, -0.18)  p<0.01* | | 0.03 (-0.17, 0.22)  p=0.78 | |
| Acronyms: β, beta coefficient; CI, confidence interval; CCBs, calcium channel blockers; mmHg, millimetres of mercury; OR, odds ratio; RASis, renin angiotensin system inhibitors; SBP, systolic blood pressure.  This table demonstrates the results of the adjusted multivariate logistic regression of ambulatory blood pressure phenotype by medication class, only using ABPM data from 1440 individuals who had ≥20 daytime readings and ≥7 nighttime readings. Similarly, this table demonstrates the results of separate adjusted logistic regression models for nocturnal hypertension and non-dipping status, in addition to separate adjusted linear regression models for 24-hour systolic and diastolic variability, with analyses limited these 1440 individuals.  *Denotes statistically significant values, using a two-sided alpha significance level of 0.05. | | | | | | | |
